# Setting the research agenda for living with and beyond cancer with comorbid illness: reflections on a research prioritisation exercise

**DOI:** 10.1186/s40900-020-00191-9

**Published:** 2020-04-29

**Authors:** D. Cavers, S. Cunningham-Burley, E. Watson, E. Banks, C. Campbell

**Affiliations:** 1 Usher Institute, University of Edinburgh, Medical School, Rm 123, Doorway 1, Teviot Place, Edinburgh, EH8 9AG UK; 2 Faculty of Health and Life Sciences, Oxford Brookes University, Jack Straws Lane, Marston, Oxford, OX3 0FL UK; 3 c/o NCRI, 2 Redman Place, Stratford, London, E20 1JQ UK

**Keywords:** Patient and public involvement, Research prioritisation, Qualitative, Living with and beyond cancer, Comorbid illness, Multi-morbidity

## Abstract

**Background:**

People living with and beyond cancer are more likely to have comorbid conditions and poorer mental and physical health, but there is a dearth of in-depth research exploring the psychosocial needs of people experiencing cancer and comorbid chronic conditions. A patient partnership approach to research prioritisation and planning can ensure outcomes meaningful to those affected and can inform policy and practice accordingly, but can be challenging.

**Methods:**

We aimed to inform priorities for qualitative inquiry into the experiences and support needs of people living with and beyond cancer with comorbid illness using a partnership approach. A three-step process including a patient workshop to develop a consultation document, online consultation with patients, and academic expert consultation was carried out. The research prioritisation process was also appraised and reflected upon.

**Results:**

Six people attended the workshop, ten responded online and eight academic experts commented on the consultation document. Five key priorities were identified for exploration in subsequent qualitative studies, including the diagnostic journey, the burden of symptoms, managing medications, addressing the needs of informal carers, and service provision. Limitations of patient involvement and reflections on procedural ethics, and the challenge of making measurable differences to patient outcomes were discussed.

**Conclusions:**

Findings from this research prioritisation exercise will inform planned qualitative work to explore patients’ experiences of living with and beyond cancer with comorbid illness. Including patient partners in the research prioritisation process adds focus and relevance, and feeds into future work and recommendations to improve health and social care for this group of patients. Reflections on the consultation process contribute to a broadening of understanding the field of patient involvement.

## Plain English summary

Little qualitative research has been done to find out what life is like for people living with and beyond cancer alongside other long term illnesses (such as heart disease or diabetes). It makes sense to ask people in this situation to have a say in issues that affect them. This paper reports on a patient involvement exercise to help identify the main things people living with and beyond cancer with other long term illness would like researchers to find out more about in order to improve their experiences. People were asked to give their views at a workshop and via an online survey. Other researchers interested in this area were also asked for their views. Five key priorities were identified: the diagnostic journey, the burden of symptoms, managing medications, addressing the needs of informal carers, and service provision. The challenges of this kind of patient involvement exercise are also discussed, focussing on ethical concerns and making discernible changes to people’s lives.

## Introduction

Approximately 363,000 people are diagnosed with a new cancer each year in the UK and the incidence is predicted to rise by a further 2% by 2035 [1]. Cancer survival has also more than doubled in the last 40 years from 24 to 50% of people living disease-free for 10 years or more as early detection and treatment improves [1]. Cancer survival is couched in the context of an ageing society, with the number of UK residents over 65 predicted to rise to 26% of the total population by 2041, compared with 15.8% in 1991. Therefore, more people are living with and beyond cancer [2]. A range of health and social care needs of people living with and beyond cancer have been described [3, 4]. However, these are often complicated by the presence of comorbid conditions. As many as 78% of cancer patients are living with at least one other condition, and the number of conditions increases as people get older [5, 6]. Cancer patients are more likely to have more comorbid conditions and therefore experience poorer physical and mental health than those without cancer [7]. There is, therefore, a need for a better understanding of the support needs of this patient group, and for service developments to better meet these needs [8].

The research agenda related to living beyond cancer has received increased attention in recent years [9], with the development of the National Survivorship Initiative [10] in England and Wales. Survivorship is also a key focus of Scotland’s Better Cancer Care [11]. A growing body of research is being developed around understanding the psychosocial dimensions of living with and beyond cancer (c.f [3, 12–15].. There have also been significant efforts to set priorities for research in this area with the National Cancer Research Institute (NCRI) and the James Lind Alliance (www.jameslindalliance.co.uk) recently reporting on the ‘Top 10 research priorities for people living with and beyond cancer’ (https://www.ncri.org.uk/lwbc/#lwbc_questions). Better coordination of care for people with complex health needs (to include multiple conditions) was identified as priority number three.

One important dimension of this field of study is the psychosocial experience of living with existing comorbid conditions alongside cancer, across the illness trajectory and beyond. At present, little has been published focusing on the experience of cancer and comorbid conditions and its implications for coordinated, quality care. A recent systematic review and evidence synthesis conducted by the authors (following PRISMA guidance) has highlighted the gaps in this field of research [16]. Themes for further exploration identified in the evidence synthesis included: *Interaction between cancer and comorbid conditions; symptom experience; illness identities and ageing; self-management, and the role of primary and secondary care.* The need for further research to understand experiences of people with cancer and other multi-morbidity has also been identified elsewhere in the literature c.f [17–20]. Further in-depth work is needed to explore what cancer means to people in relation to their other health conditions, and whether these illnesses are experienced separately or in a more holistic way. This may be less obvious to clinicians and those researching illness narratives, where the focus tends to remain on one condition. As new models of survivorship care continue to shift and accommodate patients with a complex picture of health, there are also implications for the changing roles of primary and secondary care [10, 17, 20, 21], care quality [22] and self-management [23].

### Background to patient partnership and rationale

A patient partnership model - defined here as working in collaboration with relevant patient groups as partners to develop, inform and be involved in health research, rather than a unidirectional research model with patients as *participants* whom data is collected on [24] - ensures that objectives and findings of subsequent studies are meaningful and responsive to patients’ needs, ultimately translating into relevant policy and practice [25]. Methods of stakeholder involvement in research prioritisation, including patients, is a fast developing field. In addition to more established techniques such as the Delphi method, recently applied to health care research [26, 27], a proliferation of approaches are being suggested in the literature to provide structure and transparency to the process [28]. More structured methods, such as those of the James Lind Alliance and used by the Child Health Nutrition and Research Initiative [28, 29], are expected to take precedence over the coming years. A tailored partnership approach that is patient-centred was favoured here, adapting aspects of the existing approaches to reflect the small scale of the exercise and the exploratory aims of the study [24, 30]. The approach drew on tenets of the outlined approaches, particularly the iterative workshop and consultation method used in the early phase of the Delphi approach (round one) and subsequent online questionnaires modelling the eDelphi approach (rounds two and three), also resonating with crowdsourcing techniques of consulting relevant audiences and using online methods [31] to consult with patients, their informal carers and academic experts in the field of cancer survivorship, multi-morbidity and follow-up care. A patient partnership approach is not without its challenges, however, and poses questions around purpose, effective processes and ethics that need to be addressed [32, 33].

This paper reports on small-scale research prioritisation work. It involved consulting with patients, informal carers and academic experts and combining the findings with our prior systematic review [16], to inform a follow on in-depth qualitative study exploring patient and informal carer experiences of living with and beyond cancer with comorbid illness, and health care provider experiences of caring for this patient group. This paper presents the outcomes of the research prioritisation exercise and reflects on the methodology and challenges posed by the process.

The consultation process asked:

- What are the key topics and priorities identified by individuals with a diagnosis of cancer and a comorbid illness and their informal carers?
- How can these priorities be further developed and refined with online academic expert input?
- What are the key methodological challenges posed by this research prioritisation process and what are its strengths and shortfalls?

The paper follows GRIPP2 reporting guidelines (see Additional file 1).

## Research prioritisation exercise approach

**Fig. 1:**
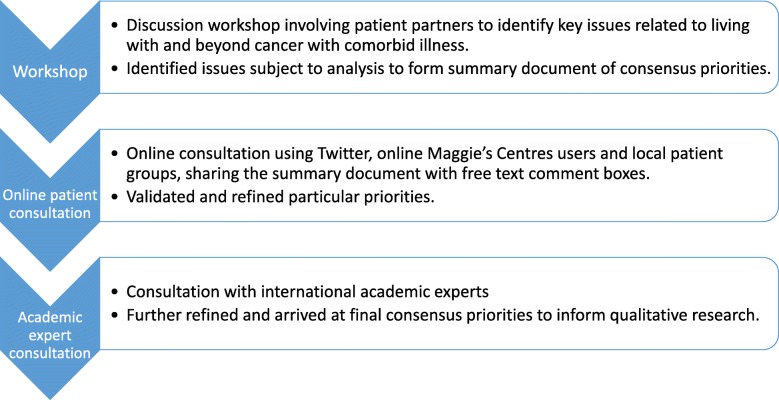
The research prioritisation process

The RPE phase of the wider project involved three components: a patient and public consultation event resulting in the development of a consultation document, online social media and email consultation with consumers on the document, and finally, consultation with academic experts to refine and finalise the document (see Fig. 1). As patient partnership engagement, this work did not require NHS ethical approval but did go through institutional ethics to review the ethical integrity of the planned approach.

### Patient and carer consultation event

People with a diagnosis of cancer and one or more comorbid illnesses and their informal carers were invited to attend a facilitated discussion workshop to share their views on key topics for research exploring the experience of living with and beyond cancer with comorbid illness. The workshop was modelled on the consultation approach in the first round of the Delphi process [28], and is a similar approach to that used previously [28, 34]. Relevant quality markers developed as part of the Delphi and CHNRI methods informed the planning and implementation of the workshop, such as adequate planning and preparation, inclusiveness of relevant stakeholders and transparency in reporting [29, 35].

Potential workshop attendees were invited via a poster advertised (either displayed in print or sent electronically) on Twitter and through the Edinburgh Cancer Centre, Edinburgh Maggie’s Centre, local GP surgeries, and consumer panels of such groups as the South East Scotland Cancer Network (SCAN) Patient Involvement Group and local charity support groups. People had the opportunity to register their interest and receive an information sheet to aid their decision to attend. Attendees were offered support with travel expenses to the central Edinburgh venue.

#### The workshop

As attendees arrived, they were offered lunch and there was informal discussion to build rapport among the group. The researcher, Debbie Cavers (DC), and consumer member of the research team Elspeth Banks (EB) then gave an introduction on the format for the day and some insights into patient and public involvement in research. It was planned that the room would then be split into small groups for in-depth discussion of ideas for research, with each group facilitated by experienced members of staff working in the field of cancer experience, as well as EB, who has considerable experience of consumer involvement and has supported similar events in the past. However, due to small numbers of attendees, only one group was formed with the facilitators. Attendees were prompted with suggested topics and questions. Post-it notes and marker pens were used to annotate ideas. With consent, the discussion was recorded using a small digital recorder to capture all thoughts for further consideration after the event. This culminated in a set of consensus priorities being documented. DC conducted further analysis of these priorities to collate and combine similar issues and summarise key patient priorities (further details below), validated by the audio recordings of the event and in the context of existing research or gaps in the field (identified by the systematic review), to form a summary of the key issues. The summary formed the basis for the consultation document shared with patients and carers online in the second part of the patient consultation process.

### Social media and email consultation of patients and carers

The summary patient priorities document, presenting the identified priorities with free text comment boxes, (modelled on the second round of an online eDelphi questionnaire approach) was shared with a group of online service users and their family members using Twitter as a platform in addition to online Maggie’s Centre and, with permission, local patient support groups, to encourage further engagement and consultation on what is relevant as a priority in the current research and policy climate. A brief outline and link to the full document (via Survey Monkey) was posted on Twitter. To reach a wide audience, relevant organisations such as the National Institute for Health Research’s INVOLVE, Our Voice Scotland, and the Alliance were tagged in order to invite their feedback. It was anticipated that the Twitter post would be shared by tagged online groups to highlight awareness of the consultation. Interested parties could post a comment with their views, or use the online link or contact the research team to request a paper copy of the consultation document with FREEPOST return envelope. Attendees at the consultation workshop were also sent the consultation document by email or post and asked for comments and feedback on each priority.

### Academic expert consultation

In order to further broaden the reach of the consultation, the document was also emailed separately to twenty professional stakeholders –academics (including clinical academics) who have published research on cancer survivorship and multimorbidity -for their input, reflective of the third round of an eDelphi online questionnaire [26]. Feedback from this exercise confirmed and added to the themes identified in the first component. These were contrasted and compared with the identified gaps in the literature from the systematic review and evidence synthesis [16]. Close parallels were drawn between the findings from the evidence synthesis and patient priorities identified via the prioritisation exercise. These were combined to inform the development of an empirical study; in particular, the research questions and the interview schedule for in-depth patient and carer interviews to be carried out by the authors.

The issues raised through the consultation were not subject to formal qualitative data analysis but did undergo an analytic process to group and summarise shared patient and academic expert priorities and link them with existing gaps in the literature identified in a separately published systematic review and evidence synthesis to produce priorities for research [16]. Issues identified at the workshop were initially added to a list. After the event, DC reviewed the list and looked for commonalities or overlap and merged points where deemed appropriate. These merged points were given overarching labels, thus generating the topics identified as priority issues. Further input from the online consultation with patients and academic stakeholders were used to refine or expand the priorities established as a result of combining the workshop and patient consultation with outputs from the systematic review, loosely following an online eDelphi approach (without explicitly ranking priorities) [26, 28]. This added to topics being expanded or new topics generated e.g. support for carers.

### Patient and public involvement

Dr. Allison Worth (PPI advisor for the Wellcome Trust Clinical Research Facility) and G.W., a Wellcome Trust consumer panel member read and commented on the proposed consultation and supporting documents (advertising poster, attendee information sheet, agenda and proposed content of discussion). The consumer member of the study advisory group, EB, supported the design and planning of the consultation event and its outcomes, including commenting on all documentation. An additional attendee at the consultation workshop was invited to join the research team as a consumer advisor for the remainder of the project.

## Outputs generated from the research prioritisation process

There were six attendees at the initial workshop to highlight topics for research, feeding into the consultation document, a much smaller number than anticipated. Due to the nature of their involvement, personal demographic information or details of their health were not recorded. However, all attendees self-reported at least one diagnosis of cancer (including bowel, breast and prostate) and one other long term condition (including heart disease, diabetes and chronic pain).

The online consultation generated a total of ten responses, again a smaller number of responses than anticipated. Of a total of 20 international academic experts contacted, eight responded or asked a member of their research team to respond on their behalf, representing countries including the UK, USA, Holland, Denmark and Australia.

Issues that can be attributed to the experience of *cancer in general* were given emphasis by those consulted, without reference to comorbid illnesses. Topics identified included: issues related to the burden of cancer-related and treatment side effects; psychological distress, anxiety, uncertainty and fear of recurrence; lack of information provision and poor communication between patient and doctors and between hospital and primary care doctors; social isolation, support and the timing of appropriate support; seeking some semblance of normality, routine and functioning in everyday life; and practical and financial concerns. The online consultation identified supported self-management, including advice and support on lifestyle and nutrition after cancer as a patient and stakeholder priority. Self-management for those with cancer and comorbid conditions was also identified as a research gap in the literature review. Research exploring the experience of cancer and comorbid illness will necessarily feature these topics and consideration is needed of how they extend to and influence living with additional chronic illness.

The key issues arising that applied to the experience of cancer and additional long term illnesses are set out below and summarised in Table 1. They have been arranged according to broad themes although many of the issues are cross-cutting.

**Table 1:** Issues identified for further qualitative inquiry

• Explore the complex symptom experience, blurring of symptoms, and the intersection with perceptions and expectations of ageing.	
• Understand help-seeking and negotiating health services for more than one illness.	
• Explore illness identity and the meaning of cancer in comparison to the other illness, with attention to issues such as cancer and other illness type, severity and sequencing.	
• Ascertain views on the notion of holistic care and continuity of care between specialists and other health and social care providers and the challenges of providing this form of care.	
• Explore preferences for treatment decision-making, professional support and follow-up, including views on specialist versus primary care.	
• Examine views on self-management and supported self-management	

### The diagnostic journey

The diagnosis of cancer was viewed as being a crucial moment in the health care trajectory and cancer journey, associated for the most part with fear, shock and distress; although in some cases possibly with relief also. Those consulted talk about their lives being turned upside down in an instant. Potential questions identified for research included a comparison of the experience of diagnosis of cancer versus another condition, with issues such as cancer and other disease type, severity and the sequencing of the illnesses being pertinent. Further consultation and analysis of feedback highlights the meaning ascribed to cancer in comparison to other conditions as being a relevant line of enquiry, with implications for illness identity.

### The burden of symptoms

Those consulted discussed the importance of looking at the impact on people’s bodies and emotions of having to deal with complex and varied symptoms of more than one condition as well as treatment side effects. It was viewed as key to explore the impact on managing daily living.

Another issue identified was the ability to make a distinction between the symptoms from the cancer or side effects of cancer treatment versus the symptoms from the other illness. A qualitative inquiry could explore any potential difference in the types of symptoms experienced and varying severity. Related to this are the varying kinds of symptoms people may experience depending on the type of cancer and the nature of their other illness. For example, for some the cancer may be treated but they are left with enduring (and/or exacerbated) symptoms from their other condition(s) or vice versa, which changes the experience of moving on from cancer. Research in this area would need to consider this carefully at the design stage. Finally, the potential blurring of symptoms - when it was not possible to know which illness was causing them; especially for general symptoms such as tiredness or dizziness – and discerning who, where and when to seek help was highlighted as a priority.

### Managing medications

A number of areas related to medications were highlighted as of potential interest for research, including managing multiple drug regimes. Management issues such as drug interactions, contraindications and their potential for unwanted side effects and emergencies (related to communication between health professionals about medications) were also raised. The impact of existing medication on side effects, treatment and trial participation were also identified in the second stage of the consultation to warrant research attention.

### Addressing the needs of informal carers

Exploration of the impact on carers was also identified as a priority in the online and academic expert consultations. This was identified as a broad and general topic to be qualitatively explored to identify carers’ unique support needs related to helping patients manage the multi-dimensional aspects of complex illness.

### Service provision

The final area highlighted as being a relevant area for research exploring the experience of cancer and comorbid illness is that of service provision. Issues of receiving good quality of care were raised and exploration of the potential tensions between the need for holistic thinking when addressing individual needs versus fragmented care were identified as a gap in the evidence base. Those consulted suggested that individuals are being followed up separately by more than one specialist and their primary care providers, and described being ‘passed from pillar to post’ with a lack of joined up care. Further qualitative investigation of this issue is required to understand more fully the experience of service provision for those with multiple conditions. Other related points identified included communication between primary and secondary care or between secondary care providers (potentially influenced by complex health conditions and an increased number of people involved in a patient’s care); the role of specialist support and reassurance (having a ‘hotline to the specialist’ versus GP care); management of follow-up for multi-morbid cancer patients; and the impact of comorbid conditions on cancer treatment decision making (as discussed in relation to managing medications). Self-management and supported self-management were also highlighted as key areas for research on patient, carer and professional views to help manage the challenges of survivorship care for people with complex health needs.

## Discussion

### Summary

Living with and beyond cancer has become a key topic of interest in cancer experience research. The added complexity of comorbid conditions among cancer survivors is one important dimension prioritised by a recent NCRI and James Lind Alliance initiative (https://www.ncri.org.uk/lwbc/#lwbc_questions). This report of a research prioritisation exercise focuses on issues relating to living with and beyond cancer with comorbid illness, tying in with priority three of the NCRI ‘top ten’.

A one-off workshop and an online consultation have combined with a systematic literature review to identify pervasive issues related to living with and beyond cancer as well as those associated with complex combinations of disease. The diagnostic journey (comparing pathways; issues of severity and sequencing of conditions), the burden of symptoms (complexity, comparison and blurring of symptoms), managing medications (polypharmacy and drug interactions), addressing carer needs (helping to manage complex illness) and issues of service provision (negotiating health services, holistic care, professional support and follow-up and supported self-management) have all been identified as areas where more exploratory research is needed to understand people’s views and experiences in these circumstances.

This exercise, combined with a systematic review of the literature that has identified gaps in the qualitative evidence base that map onto patient-identified priorities [16], has highlighted key issues (as listed above) that appear to be central to the experience of cancer with additional conditions. This approach, working in partnership with people living with and beyond cancer with comorbid conditions, has identified priorities for future empirical research.

### Reflections on the research prioritisation process

A patient partnership and stakeholder involvement approach was adopted to plan meaningful and relevant research to ultimately influence policy and practice and improve care for patients and their families. However, it is important to consider one’s own assumptions in influencing interpretations of priorities in order to fit in with planned research. Practicing reflexivity is key and keeping an open agenda for topics to be added to can help to address these concerns [36].

Going into this exercise, the research team held certain assumptions that is it important to surface here and consider the extent to which they were challenged. The team are interested in psychosocial aspects of living with and beyond cancer and so cancer was the index condition in relation to other comorbid illness. Therefore, cancer has already been implicitly prioritised as the centre of patients’ stories. Hearing suggestions of illness identity and prioritisation of conditions as being more complex, and cancer not always being at the centre of people’s lives (depending on the type and severity of the cancer and comorbidities), has challenged this assumption.

There can also be a mismatch between patients’, clinicians’ and research community priorities for research, so taking a partnership approach involving the views of all of these groups was important to inform the priorities set and shape future work [37]. There is an additional danger of a ‘policy disconnect’ [as described by Alan Irwin in his critical review of public involvement in relation to science and technology research [38]], where there is a lack of evidence that patient partnership work is taken forward and leads to tangible changes in care provision. It can often be difficult to see the thread from prioritisation and then research through to improved care and experience, particularly in studies exploring more conceptual dimensions of living with illness. The steps in providing the evidence base are often slow from early consultation and exploratory work, to more focused topic work before reaching practical interventions that may be shown to improve patient care. Embedding these in sustained provision of care in a resource constrained National Health Service is yet another challenge.

### Limitations of the exercise

Ultimately, this consultation did not generate as much involvement and feedback as was anticipated and planned for by the research team, impacting on confidence in the consensus reached. Although limited by time and financial resources, there are important lessons to be learned about mitigation planning and a flexible approach to enable expansion on consultation methods in response to low interest, as set out in the Delphi approach [28]. People’s motivations for patient involvement are complex [39]. Upon reflection, it is possible that ill health and limited mobility prevented people from becoming involved, which will have included travel for some. There are also possible social or structural barriers: perhaps people did not feel they qualified to represent others in this way or comfortable working in an academic environment on such a project? It certainly felt as though there was more groundwork to be done in terms of building productive relationships and allowing people time to share their personal stories before moving forward to a shared research agenda, suggesting that a longer term process may have generated different, and possibly better, outcomes. To this end, a second workshop after the online consultation would have helped to consolidate or expand on the priorities identified in the initial workshop and reach a clearer consensus, but resource and time constraints did not permit this.

There are also limitations to the number of people taking part in the consultation and reflections to be made on the planning process to ensure a broader scope and diversity of consultants, speaking to existing debates around whether or not patient involvement includes the voices of a wide enough group of people, including those from marginalised groups and those less able to expertly self-advocate and articulate their views and beliefs [32]. This brings into question whether formal training should be offered to more people in order to empower and enable people of all backgrounds to engage with the involvement process more fully [40]. This sits within a wider critical debate about the role of and spaces occupied by participatory research [33, 40] and potential power imbalances in the context of citizen science and consumer accountability [41–43]. The importance of bringing in evidence relating to gaps in the literature identified in the aforementioned systematic review carried out by the authors, as well as subsequent empirical research to capture the diverse needs of patients living with and beyond cancer with comorbid conditions, is also heightened, to contribute to a robust evidence base with transferable findings.

DC is also a relative novice in doing this form of patient involvement work and so there were lessons to be learned in terms of publicising the event and online consultation, as well as a lack of power and influence in reaching certain groups and being heard. Approaches where invitations came from a trusted source, e.g. posters in clinic rooms or personal invitations endorsed by online patient group coordinators, appeared to be more successful than social media appeals with endorsement of trusted sources. Employing a wider range of strategies for finding people in order to invite them to be involved in this consultation, such as via community groups and councils, may have been a better approach [44].

This research prioritisation exercise did pose some challenging questions around the blurring of the boundaries between patient involvement work and research. It problematises what counts as research data and therefore how the study should be designed and the ethical considerations managed, including the role of formal ethical review (procedural ethics). These issues seem particularly pertinent in relation to scenarios such as this exercise where information is being generated that will be used to support research and policy impact. The line between treating the discussion as informing research and as research data can be blurred: this paper is an example of that ambiguity. However, important steps have been taken to clarify this distinction, both here and in the broader literature, and a call for transparency and clarity in reporting of engagement work [45]. The engagement process employed a sensitive and considered ethical approach and went through required institutional approval (Usher Research Ethics Group). It was clear that people were being asked to join the researchers as partners in a process of collaborative information collection, and the discussions were summarised and key issues identified, rather than analysed as they would be in a qualitative research process. However, a social constructionist approach to intersubjective data generation is arguably not that different; neither is a thematic analysis. While we feel confident in our own ethical position, we are nonetheless uncomfortable operating within the ambiguous space of patient involvement in research that is, if it is to be effective, generative of relevant information in a research context. We would argue that a new approach to ethics is needed in this environment to work with this tension and to reassure both researchers and patient participants that an appropriate form of ethical review has been undertaken and that ethical issues will continue to be reflected upon during the process of the engagement and subsequent research. This is all the more important as PPI is increasingly a core component of research in the UK [46].

This consultation did not undergo formal evaluation but it was clear that those involved helped to validate the thinking of the research team and contributed topics that would not otherwise have been identified without consulting stakeholders. We would argue that more attention needs to be paid to evaluation, including by self-reflection, and for this to contribute to the evidence base for PPI.

## Conclusion

Including patients in the consultation in addition to academics and clinicians with a broad view of the research landscape on an international stage aims to lead to more meaningful and relevant in-depth research and, ultimately, to corresponding interventions that will improve patient, carer and provider experience.

Findings from this research prioritisation exercise will directly inform further in-depth research exploring experience of cancer and other comorbid conditions as topics identified will be integrated into a planned interview study with patients and carers. On a broader scale, reflections on the consultation will contribute to understandings of the role of patient partners in the research process. It can provide the foundations for future work to provide outputs that are relevant and meaningful, not only to patients, but to policy makers, in order to make recommendations for health and social care services in Scotland, as elsewhere, and improve patient experience, to meet the targets set out by Better Cancer Care [11].

## Supplementary information

**Additional file 1.**

## Data Availability

This paper does not include research data and this statement is therefore not applicable.

## Abbreviations

NCRI: National Cancer Research Institute
PPI: Patient and Public Involvement
DC: Debbie Cavers
EB: Elspeth Banks
GP: General Practitioner
NHS: National Health Service
CSO: Chief Scientist’s Office
