# Supplementary material for: Setting the research agenda for living with and beyond cancer with comorbid illness: reflections on a research prioritisation exercise
Source: Res Involv Engagem. 2020 Apr 29;6:17. doi: 10.1186/s40900-020-00191-9 (PMC7191759; doi:10.1186/s40900-020-00191-9)
Supplement: Supplementary file 1 — Additional file 1. [file 40900_2020_191_MOESM1_ESM.docx]

Cookie Notice

We use cookies to personalise content and ads, to provide social media features and to analyse our traffic. We also share information about your use of our site with our social media, advertising and analytics partners in accordance with our [Privacy Statement](https://www.biomedcentral.com/privacy-statement). You can manage your preferences in 'Manage Cookies'.

Close

OK

Manage Cookies

- Your Privacy
- Strictly Necessary Cookies
- Performance & Analytics Cookies
- Functional Cookies
- Targeting Cookies
- [More Information](https://www.biomedcentral.com/cookies)

Privacy Preference Centre

Top of Form

**Active**

Bottom of Form

**Always Active**

GRIPP2 Short form

| **Section and topic** | **Item** | **Reported on page No** |
| --- | --- | --- |
| 1: Aim | Report the aim of PPI in the study | 5 |
| 2: Methods | Provide a clear description of the methods used for PPI in the study | 6-8 |
| 3: Study results | Outcomes—Report the results of PPI in the study, including both positive and negative outcomes | 8-12 |
| 4: Discussion and conclusions | Outcomes—Comment on the extent to which PPI influenced the study overall. Describe positive and negative effects | 12-16 |
| 5: Reflections/critical perspective | Comment critically on the study, reflecting on the things that went well and those that did not, so others can learn from this experience | 13-16 |
